# Optimizing head movement patterns to maximally modulate CSF flow by myodural bridge complex

**DOI:** 10.1097/MD.0000000000045463

**Published:** 2025-10-24

**Authors:** Yang Song, Hong-Jin Sui, Jian-Fei Zhang, Ji-Hang Li, Yi-Tong Sun, M. Adeel Alam Shah, Chan Li, Campbell Gilmore, Wen-Bin Jiang

**Affiliations:** a Department of Anatomy, College of Basic Medicine, Dalian Medical University, Dalian, China; b School of Management, Liaoning University of International Business and Economics, Dalian, China; c Department of Radiology, Dalian University Affiliated Xinhua Hospital, Dalian, China; d Department of Anatomy, Shantou University Medical College, Shantou, China; e Medical School, St. George’s University of London, London, United Kingdom.

**Keywords:** CSF, headache, myodural bridge complex, neurodegenerative diseases

## Abstract

The myodural bridge complex (MDBC) is a tendon-like structure highly conserved during vertebrate evolution, suggesting it plays an important physiological role. Substantial evidence indicates that the MDBC may contribute to cerebrospinal fluid (CSF) circulation by generating mechanical force. However, the proprioceptive innervation within the MDBC remains poorly characterized. This study aimed to systematically identify the types of sensory nerve endings within the MDBC, thereby providing a neuroanatomical basis for optimizing mechanostimulation and developing new therapeutic strategies for neurodegenerative diseases arising from faulty CSF clearance. Tissue blocks containing the suboccipital region were harvested from 4 donated adult cadaveric specimens. The samples were embedded in paraffin, and histological sections were prepared. Immunohistochemical staining for S100 (using a kit protocol) was performed to identify neural structures. Receptor types and distributions within the MDBC were identified using light microscopy based on established morphological criteria. Findings were reported using descriptive analysis. Four types of sensory nerve endings were embedded within MDBC fibers: Type I: Ruffini corpuscles, predominantly distributed perivascularly with adjacent MDBC fibers; Type II: Pacinian corpuscles were found near the spinal dura mater and the rectus capitis posterior minor muscle, located within loose connective tissue next to the MDBC fiber bundles; Type III: Golgi-Mazzoni corpuscles, observed infrequently only at the ventral aspect of the rectus capitis posterior minor muscle near the atlas; Type IV: Free nerve endings, widely distributed among MDBC structures. Within MDBC connective tissue, Ruffini corpuscles, Pacinian corpuscles, and free nerve endings predominate, while Golgi-Mazzoni corpuscles exhibit sparse distribution. This receptor profile suggests that progressive compound head movements coupled with suboccipital vibratory stimulation are likely to optimally activate mechanical transmission within the MDBC, thereby efficiently enhancing CSF siphoning.

## 1. Introduction

Cerebrospinal fluid (CSF) is the primary component of the extracellular fluid in the central nervous system. Recent studies have shown that CSF nourishes neural tissue and removes metabolic waste from the brain.^[1]^ Its flow is modulated by arterial pulsation, respiratory movements, body position, and blood circulation.^[2]^ Recent breakthroughs in neuroscience demonstrate that inadequate clearance of metabolic waste by the CSF may contribute to neurodegenerative diseases, especially Alzheimer disease, expanding our understanding of the CSF system’s regulatory role.^[3,4]^ However, the dynamics of CSF remain incompletely understood. A deeper understanding of the physiological mechanisms regulating CSF flow may provide significant insights for diagnosis and treatment of neurodegenerative diseases.^[5]^ Emerging evidence indicates that mechanical stimulation of superficial lymphatic vessels in the head and neck significantly enhances CSF drainage, establishing a foundation for novel therapeutic approaches to neurodegenerative disorders.^[6]^ Nevertheless, a similar phenomenon is also observed in the suboccipital region.

Studies have demonstrated that the suboccipital region contains fibroconnective tissue bridges originating from the obliquus capitis inferior muscle, rectus capitis posterior major muscle (RCPma), rectus capitis posterior minor muscle (RCPmi), and nuchal ligament.^[7]^ These bridges traverse the posterior atlanto-occipital interspace (PAOiS) and posterior atlanto-axial interspace to connect with the posterior atlanto-occipital membrane, vertebral dura ligament, and upper cervical spinal dura mater (SDM), constituting the myodural bridge complex.^[8–10]^ Recent research has established that myodural bridge complex (MDBC) is phylogenetically conserved across vertebrate species.^[10]^ During head movement, this highly conserved structure has been shown to exert tension on the SDM, alter local subarachnoid space pressure, and generate pump-like propulsion for CSF circulation.^[5,11–15]^ Clinical studies have further indicated that mechanical stimulation of the suboccipital region effectively modulates symptoms in patients of Parkinson disease.^[16]^ Moreover, such stimulation has demonstrated therapeutic efficacy in headache management^[17]^ and hamstring shortening syndrome intervention,^[18]^ effects potentially mediated through MDBC-facilitated CSF siphoning.^[17,18]^ Therefore, identifying the head movement patterns that most effectively stimulate MDBC mechanical transmission may have significant implications for treating associated pathologies.

Scali et al,^[19]^ identified proprioceptive nerve endings within the MDB structures between the RCPma and SDM in the posterior atlanto-axial interspace. They proposed that these proprioceptors facilitate neural transmission and provide feedback on SDM tension during normal head and neck movement. However, no published data exist regarding proprioceptive innervation within MDB between the RCPmi and SDM in the PAOiS. Zheng et al,^[9]^ postulated that MDB exerts significant cooperative effects during both head movement and static positioning. Consequently, the MDB likely contributes not only to biomechanical support but also to proprioception, functioning synergistically with the suboccipital muscles to maintain equilibrium and regulate cephalic kinesthesia. Freeman et al,^[20]^ classified sensory nerve endings into 4 types: Ruffini corpuscles, Pacinian corpuscles, Golgi-Mazzoni corpuscles, and free nerve endings, each responding to specific mechanical stimuli.

Conversely, an investigation into suboccipital musculature and head movement revealed that only the cross-sectional area of the RCPmi at the atlanto-occipital joint positively correlated with head movement stability,^[21]^ highlighting the unique role of this muscle in postural control. Therefore, comprehensive characterization of the sensory nerve ending types and their distribution within the MDB in the PAOiS is essential to better understand head movement patterns that optimally stimulate MDB-mediated mechanical transmission. This understanding may provide novel perspectives for clinical headache management and inform therapeutic interventions for neurodegenerative disorders.

## 2. Materials and methods

### 2.1. Ethics statement

This study received approval from the Ethics Committee of the Dalian Medical University (20180306). The investigation utilized 4 head and neck specimens from adult Chinese donors sourced through the Body and Organ Donation Center. Written informed consent was obtained from all donors prior to death in accordance with Ethics Committee regulations.

### 2.2. Tissue harvesting

Four adult head and neck specimens were utilized and fully-fixed in 10% formaldehyde solution for about 4 weeks. Following complete fixation, a T-shaped incision was made in the occipital region: a transverse incision of about 12 cm along the superior margin of the external occipital protuberance bilaterally, with a midline longitudinal incision of about 12 cm extending from its midpoint. Superficial muscles and soft tissues were removed to expose the RCPma and OCI. These muscles were then traced to their origin on the spinous process of the axis (C2). The axis vertebra position was identified, and tissue blocks approximately 12 cm × 12 cm were harvested using oscillating and band saws. These blocks were demarcated superiorly by the superior nuchal line of the occipital bone and laterally by the bilateral borders of the RCPma, surrounding suboccipital muscles, posterior arch of atlas, lamina of axis, and posterior SDM. Subsequent removal of occipital bone, atlas, and axis vertebrae was performed. Specimens underwent 12 to 24 hours of running-water rinsing before standard dehydration, clearing, and paraffin embedding. Embedded tissue blocks were sectioned at 8 to 10 μm thickness.

### 2.3. Immunohistochemical staining

Immunohistochemical analysis was performed using a commercially available ready-to-use kit for S100 (Beijing Bioss Biological Technology Co., Ltd.). According to the instructions provided by the manufacturer, sections underwent routine dewaxing and hydration, followed by three 5 minutes phosphate-buffered saline (PBS) washes. Endogenous peroxidase activity was blocked by 3% H_2_O_2_ at room temperature for 20 minutes. After 3 additional 5 minutes PBS washes, nonspecific binding sites were blocked with normal goat serum (20 minutes incubation). Primary antibody against S100 protein (1:1000 dilution; Abcam ab52642, UK) was applied, which specifically binds S100 protein in Schwann cells. Sections were incubated at 4 °C for 24 hours. Following three 5 minutes PBS washes, biotinylated secondary antibody (goat anti-mouse/rabbit IgG peroxidase) was applied for 20 minutes. After 3 further 5 minutes PBS washes, sections were incubated with horseradish peroxidase-conjugated streptavidin working solution (20 minutes). Subsequent to 3 final 5 minutes PBS washes, 3,3′-diaminobenzidine chromogenic development was performed. Sections were rinsed under running tap water, counterstained with hematoxylin, dehydrated, and mounted with neutral gum. To confirm reaction specificity, negative control sections processed without primary antibody underwent identical treatment. Results were examined by light microscopy.

## 3. Results

**Figure 1.**
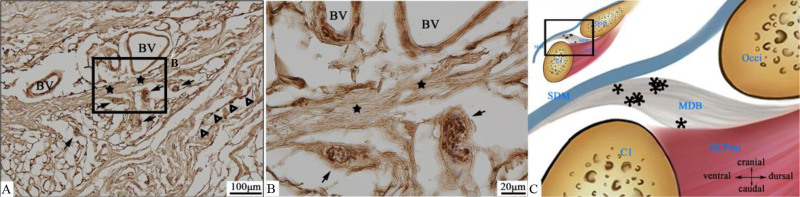
Ruffini corpuscles (tailed arrow →) and free nerve endings (hollow triangle △). (A) At 10× magnification, multiple Ruffini corpuscles (tailed arrows →) distributed perivascularly with traversing collagen fiber bundles (pentagram ★). (B) Higher magnification of boxed area in (A), (40×). Pacinian corpuscle (tailed arrow →) enclosed by thin capsule, clearly showing internal neural branches and terminals. (C) Schematic diagram: Location of Ruffini corpuscles (asterisk *) within MDB. BV = blood vessel, C1 = atlas, MDB = myodural bridge, Occi = occipital bone, RCPmi = rectus capitis posterior minor muscle, SDM = spinal dura mater.

Results demonstrated widespread distribution of free nerve endings (Fig. 1A) throughout connective tissue within the MDB across all specimens in the PAOiS, though with inter-specimen variation and differential distributions among receptor types.

Ruffini corpuscles were observable in most sections. These receptors displayed perivascular localization with traversing collagen fibers (Fig. 1A). A thin capsule enveloped the peripheral structure, permitting clear visualization of internal neural branches and terminals connecting with adjacent collagen fibers (Fig. 1B).

**Figure 2.**
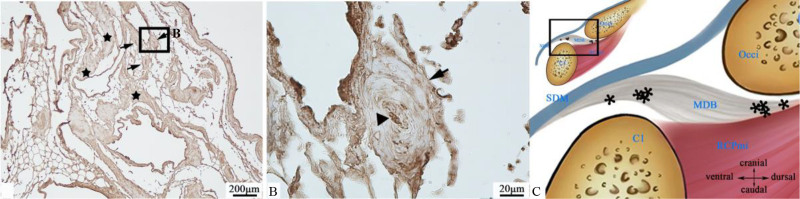
Distribution of Pacinian corpuscles (tailed arrow →). (A) At 4× magnification, Pacinian corpuscle (tailed arrow →) embedded within loose connective tissue with traversing collagen fiber bundles (pentagram ★). (B) Higher magnification of boxed area in (A), (40×). Concentric lamellar structure encloses deeply stained inner core (tailless arrow ▲). Lamellar cells exhibited no immunoreactivity to S100 protein antibody. (C) Schematic diagram: Location of Pacinian corpuscles (asterisk *) within the MDB. C1 = atlas, MDB = myodural bridge, Occi = occipital bone, RCPmi = rectus capitis posterior minor muscle, SDM = spinal dura mater.

Pacinian corpuscles were clearly identified in the adult specimen. These receptors embedded within loose connective tissue between the MDB fibers, with collagen fiber bundles traversing adjacent regions (Fig. 2A). The corpuscles displayed characteristic concentric lamellae formation, exhibiting an ovoid onion-like configuration in cross-section with approximate diameter of 60 μm (Fig. 2B). Immunohistochemical analysis revealed strong S100 protein positivity in the inner core lamellae, particularly within the central axon compartment.

**Figure 3.**
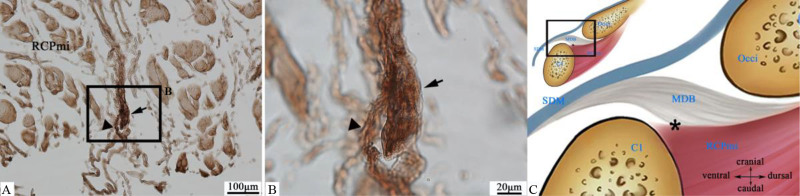
Distribution of Golgi-Mazzoni corpuscles (tailed arrow →). (A) At caudal aspect of rectus capitis posterior minor muscle (RCPmi), Golgi-Mazzoni corpuscle (tailed arrow →) within intermuscular fascia. Penetrating neural branches (tailless arrow ▲) visible, (4×). (B) Higher magnification of boxed area in (A), (40×). Magnified Golgi-Mazzoni corpuscle (tailed arrow →) demonstrating elongated morphology. Neural terminals (tailless arrow ▲) penetrate corpuscle with arborizing branches forming terminal expansions. (C) Schematic diagram: Location of Golgi-Mazzoni corpuscles at junction between the RCPmi and the MDB near atlas (asterisk *). C1 = atlas, MDB = myodural bridge, Occi = occipital bone, RCPmi = rectus capitis posterior minor muscle, SDM = spinal dura mater.

Golgi-Mazzoni corpuscles were infrequently observed in 4 specimens, identified in the near atlanto-axial joint at the caudal aspect of the RCPmi. This receptor type resided within intermuscular connective tissue, featuring a thin capsule enveloping neural terminals that formed elongated expansions (Fig. 3).

## 4. Discussion

The MDBC represents a phylogenetically conserved fibroconnective structure in vertebrates,^[10]^ shown to play a role in modulating CSF circulation.^[5,11–15]^ A comprehensive characterization of sensory nerve ending types and distributions within this complex will elucidate head movement patterns that optimally activate MDBC mechanical transmission. This understanding may provide novel therapeutic perspectives for clinical headache management and treatment for neurodegenerative diseases.

Consistent with Freeman classification of sensory nerve endings,^[20]^ 4 mechanoreceptor types were identified within the MDB fibers. Type I: Ruffini corpuscles. Predominantly distributed perivascularly with adjacent MDB fibers. These receptors detect static joint position, internal pressure, and dynamic movement parameters including angle, velocity, and angular acceleration.^[22]^ Type II: Pacinian corpuscles, located near the SDM and the RCPmi, embedded within loose connective tissue alongside traversing MDB fiber bundles. They mediate kinesthesia and respond to high-frequency vibration and acceleration changes.^[23]^ Type III: Golgi-Mazzoni corpuscles, observed infrequently, exclusively at the ventrocaudal aspect of the RCPmi near the atlas. These receptors demonstrate minimal responsiveness during standard joint motion but exhibit high sensitivity to end-range tensile forces.^[24]^ Type IV: Free nerve endings, widely distributed throughout MDB, detecting inflammatory and nociceptive stimuli.^[20]^

The widespread distribution of Ruffini corpuscles (type I) indicated their high sensitivity to low-speed sustained traction. Combined with their functional capability to perceive static joint position and dynamic parameters (angle/velocity), this demonstrates that slow flexion–extension compound rotation movements could effectively stimulate mechanical transmission of the MDBC through continuous tension changes, promoting rhythmic traction of the SDM. The enrichment of Pacinian corpuscles (type II) near the SDM and muscle bellies reflected their responsiveness to high-frequency vibration and acceleration changes. This provided theoretical support for applying vibration therapy to the suboccipital region. Targeted vibration might activate Pacinian corpuscles to enhance the instantaneous traction effect of MDBC on the SDM, thereby amplifying CSF siphoning. The scarcity of Golgi-Mazzoni corpuscles (type III) carried important warning significance. As these structures served as “safety sentinels” at extreme joint positions, their rare distribution within MDBC suggests that vigorous head movements, particularly whiplash injuries, could easily breach the mechanical protection threshold of the atlanto-occipital joint, leading to MDBC fiber tearing and the SDM tension imbalance. From a neuroanatomical perspective, this finding explains why sudden hyperextension or hyperflexion of the head and neck should be clinically avoided.^[21]^

The extensive presence of free nerve endings (type IV) revealed the nociceptive function of MDBC. When activated by inflammation or abnormal mechanical loading, these endings might inhibit head movement through spinal cord–brainstem reflex pathways, thereby indirectly limiting the regulatory capacity of MDBC for CSF flow. This mechanism provided a novel explanation for head–neck movement restriction commonly observed in patients with chronic headache.^[25]^

This study further validated hypotheses of Scali^[19]^ and Zheng et al^[9]^: MDBC functions not only as a mechanical transmission bridge but also as a functional complex integrating proprioception and mechanical transmission. This dual attribute established new pathways for suboccipital intervention strategies. Mechanical transmission pathway: Low-speed sustained compound movements (e.g., progressive head flexion–extension with 10–15 axial rotations) maximized utilization of mechanical sensitivity of Ruffini corpuscles. This achieved rhythmic traction of the SDM and enhanced CSF siphoning. This also explained the superior therapeutic efficacy of compound head movements in tension-type headache intervention compared to other exercise modalities.^[17]^ Proprioception pathway: The vibration sensitivity of Pacinian corpuscles allows for non-displacement stimulation, such as vibratory input applied to the skin over the posterior atlanto-occipital interspace, to potentially activate proprioceptive pathways. This stimulation may directly modulate SDM tension, offering an alternative therapeutic approach for patients with restricted head and neck movement. Additionally, it may provide novel insights into the mechanisms underlying the reported efficacy of suboccipital acupuncture in patients with Parkinson disease.^[16]^

Previous research has demonstrated that vibratory stimulation of the superficial lymphatic vessels in the head and neck significantly enhances CSF drainage.^[6]^ However, in comparison, mechanical transmission mediated by the MDBC during head movement may exert a more direct influence on intracranial structures, generating a siphoning effect that facilitates CSF circulation. This mechanism warrants further experimental validation. Recent research has shown that neurodegenerative diseases, such as Alzheimer, may originate from impaired metabolic waste clearance by the CSF.^[3,4]^ Regulation of CSF dynamics via the MDBC presents a promising therapeutic target for neurodegenerative conditions such as Parkinson disease and Alzheimer disease. Future investigations could explore the development of suboccipital directional exercise regimens in combination with low-frequency vibratory stimulation applied to the suboccipital region. This approach aims to activate proprioceptive pathways through non-displacement stimulation, leveraging the vibration sensitivity of mechanoreceptors such as Pacinian corpuscles. To ensure safety and efficacy, such interventions should avoid rapid or whiplash-like movements and instead employ gradually progressive tension-loading protocols. These precautions are essential to prevent reflexive movement inhibition mediated by nociceptive free nerve endings.

In addition to the use of partially archived specimens, this study has several limitations that should be considered. First, the sample size was small, which, while sufficient for an initial qualitative histological investigation, may limit the generalizability of the findings and preclude meaningful statistical analysis of receptor distribution patterns. Second, although the core histological processing protocols (e.g., fixation, embedding, and staining) were standardized, minor variations in the initial specimen harvesting and fixation durations were inevitable due to the nature of the donated samples. Finally, detailed donor information, including precise age, cause of death, and comprehensive medical history (particularly regarding neurological or musculoskeletal disorders), was unavailable. These factors could potentially influence the integrity and density of the observed neural structures. Future studies utilizing larger, prospectively collected cohorts with well-documented donor characteristics are warranted to confirm and expand upon these initial observations.

## 5. Conclusion

This study identified 4 types of neural mechanoreceptors within the MDBC connective tissue. Type I: Ruffini corpuscles: Predominantly distributed perivascularly; Type II: Pacinian corpuscles: Embedded within loose connective tissue; Type III: Golgi-Mazzoni corpuscles: Infrequently observed; Type IV: Free nerve endings: Most abundant type. The presence and distribution of these mechanoreceptors provide a kinesiological basis for developing clinical intervention targeting headaches and neurodegenerative disorders. Specifically, therapeutic strategies incorporating progressive compound head movements in conjunction with suboccipital vibratory stimulation may modulate MDBC-mediated proprioceptive and CSF regulatory functions.

## Acknowledgments

We thank large language models DeepSeek R1 model (https://chat.deepseek.com/) for the revision of this manuscript language.

## Author contributions

**Conceptualization:** Wen-Bin Jiang.

**Data curation:** Yang Song, Wen-Bin Jiang.

**Formal analysis:** Yang Song, Wen-Bin Jiang.

**Funding acquisition:** Hong-Jin Sui.

**Investigation:** Yang Song, Jian-Fei Zhang, Ji-Hang Li, Yi-Tong Sun, Wen-Bin Jiang.

**Methodology:** Yang Song, Jian-Fei Zhang, Wen-Bin Jiang.

**Software:** Yang Song, Wen-Bin Jiang.

**Supervision:** Hong-Jin Sui, Wen-Bin Jiang.

**Validation:** Wen-Bin Jiang.

**Visualization:** Wen-Bin Jiang.

**Writing – review & editing:** Hong-Jin Sui, Ji-Hang Li, Yi-Tong Sun, M. Adeel Alam Shah, Chan Li, Campbell Gilmore, Wen-Bin Jiang.

**Writing – original draft:** Yang Song, Wen-Bin Jiang.
